# *EGFR*突变非小细胞肺癌患者奥希替尼诱导间质性肺疾病后奥希替尼再挑战：病例报道

**DOI:** 10.3779/j.issn.1009-3419.2021.102.39

**Published:** 2021-11-20

**Authors:** 俊杰 谷, 帆 白, 兰 宋, 颖轶 王

**Affiliations:** 1 100730 北京，中国医学科学院北京协和医院内科 Department of Internal Medicine, Peking Union Medical College Hospital, Beijing 100730, China; 2 100730 北京，中国医学科学院北京协和医院药剂科 Department of Pharmacy, Peking Union Medical College Hospital, Beijing 100730, China; 3 100730 北京，中国医学科学院北京协和医院放射科 Department of Radiology, Peking Union Medical College Hospital, Beijing 100730, China; 4 100730 北京，中国医学科学院北京协和医院肿瘤内科 Department of Oncology, Peking Union Medical College Hospital, Beijing 100730, China

**Keywords:** 表皮生长因子受体, 间质性肺疾病, 肺肿瘤, 奥希替尼, Epidermal growth factor receptor, Interstitial lung disease, Lung neoplasms, Osimertinib

## Abstract

奥希替尼诱导的间质性肺疾病（interstitial lung disease, ILD）是一种罕见的、致命的肺毒性疾病。我们报道1例64岁男性IV期肺腺癌患者，伴有表皮生长因子受体（epidermal growth factor receptor, *EGFR*）外显子19缺失，使用奥希替尼80 mg/d作为一线靶向治疗。奥希替尼开始治疗后第60天患者出现ILD。立即停用奥希替尼，并开始口服泼尼松60 mg/d，ILD在13 d内得到改善。权衡风险和获益后，再次开始奥希替尼与泼尼松治疗。奥希替尼治疗16个月以上，患者既没有疾病进展，也没有ILD复发。根据我们的病例和既往文献，在仔细评估*EGFR*突变非小细胞肺癌（non-small cell lung cancer, NSCLC）患者的风险及获益后，在类固醇激素辅助下再次使用奥希替尼可被视为一种有效的治疗选择。

## Abstract (en)

Osimertinib-induced interstitial lung disease (ILD) is an uncommon, but fatal pulmonary toxicity in some patients. We report a case of a 64-year-old male with stage IV adeno-non-small cell lung cancer (NSCLC) harboring an exon 19 deletion in the epidermal growth factor receptor (*EGFR*) treated with osimertinib 80 mg/d for first-line targeted therapy. On day 60 after initiating treatment of osimertinib, the patient developed ILD. Osimertinib was discontinued immediately and oral prednisone 60 mg/d was initiated, ILD improved within 13 d. After balancing the risk and benefit, osimertinib was restarted concurrently with prednisone. The patient showed neither disease progression nor a recurrence of ILD for more than 16 months. Based on our case and literature review, retreatment with osimertinib under steroid coverage could be considered as an effective treatment option after careful risk-benefit assessment for patients with *EGFR*-mutant NSCLC.

作为第三代表皮生长因子受体酪氨酸激酶抑制剂（epidermal growth factor receptor-tyrosine kinase inhibitors, EGFR-TKIs），奥希替尼（AZD9291）已被批准用于一线治疗*EGFR*外显子19缺失或外显子21 L858R突变的转移性非小细胞肺癌（non-small cell lung cancer, NSCLC）患者。

奥希替尼诱导的间质性肺疾病（interstitial lung disease, ILD）并不常见，但在一些患者中可能危及生命。据报道，奥希替尼诱导的间质性肺病发生率为2%-3%^[1-3]^，约15%的病例是致命性的，死亡率约为1.5%。

一旦ILD导致奥希替尼停用，随后即使ILD恢复，通常不建议再次使用奥希替尼。根据AURA3试验^[4]^，尽管不如再次使用EGFR-TKIs有效，大多数患者随后接受化疗。因此问题在于奥希替尼再挑战是否仍能使患者获益。尽管很多文献均有报道吉非替尼、厄洛替尼或奥希替尼成功再挑战，但奥希替尼再挑战是否合理仍未达成共识。本研究中我们描述了1例*EGFR*突变的晚期NSCLC患者，接受一线奥希替尼治疗后发生了ILD，随后停用奥希替尼，待ILD恢复后再次使用奥希替尼。

## 1 病例报道

该病例是1例64岁的中国男性，不吸烟，确诊为IV期肺腺癌，伴有*EGFR*敏感突变（外显子19缺失）。2018年7月开始奥希替尼（80 mg, *qd*）一线靶向治疗（图 1A），2018年8月评估为部分缓解。奥希替尼治疗后的第60天，该患者出现呼吸困难和干咳。胸部计算机断层扫描（computed tomography, CT）示右肺新发散在磨玻璃影，左上肺多发实变、结节、磨玻璃影、纤维索条影（图 1B）。这些病变影像学上符合奥希替尼诱发的ILD。治疗上立即停用奥希替尼，同时开始口服泼尼松（60 mg, *qd*）。给药后第13天，呼吸困难减轻，肺部病变改善（图 1C）。由于临床认为标准化疗不可行，2019年2月奥希替尼（80 mg, *qd*）与泼尼松（30 mg, *qd*）同时重新给药，ILD未复发，病情稳定（图 1D）。随后泼尼松逐渐减少到每天3.75 mg。患者随后成功接受奥希替尼治疗16个月以上，无疾病进展或ILD复发。

**图 1:**
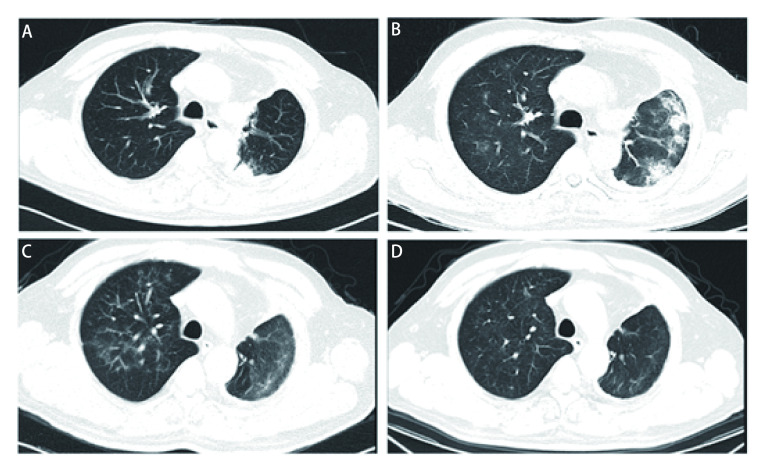
胸部CT（肺窗）。A：奥希替尼治疗前，基线CT显示左上叶胸膜下肺病变；B：奥希替尼治疗60 d后，CT显示左肺上叶多发实变、结节、磨玻璃影、纤维索条影较图1A明显，右肺出现新的散在磨玻璃影；C：使用泼尼松治疗后13 d，CT显示上述病变明显缓解；D：奥希替尼再挑战10个月后，CT未见ILD复发征象。 Chest CT of lung window. A: Before treatment with osimertinib, the baseline CT imaging showed subpleural lung lesion in the left upper lobe; B: Sixty days after the initiation of osimertinib, the CT imaging showed multiple consolidations, nodules, ground glass opacities (GGOs), and fibrous cables in left upper lobe, significantly remarkable than Fig 1A and new scattered GGOs in the right lung; C: Thirteen days after the administration of prednisone, the CT imaging showed remarkable remission of these lesions; D: Ten months after osimertinib rechallenge, the CT imaging showed no recurrence of ILD. CT: computed tomography.

## 2 讨论

根据FLAURA试验，奥希替尼是一个口服的有效的第三代EGFR-TKI，相比于第一代及第二代EGFR-TKIs表现出明显优越的疗效，无进展生存期（progression-free survival, PFS）（18.9个月*vs* 10.2个月）和总生存期（overall survival, OS）（38.6个月*vs* 31.8个月）^[5, 6]^。基于AURA3和FLAURA试验，美国国家综合癌症网络（National Comprehensive Cancer Network, NCCN）指南考虑将奥希替尼作为*EGFR*外显子19缺失或外显子21 L858R突变的NSCLC患者的一线靶向治疗。

尽管奥希替尼通常耐受性良好，但在一些患者中，ILD虽不常见却是一种致命的肺部并发症^[7]^。ILD的机制尚不明确。EGFR在II型肺上皮细胞上表达，并参与肺泡壁修复。EGFR-TKIs通过中断肺泡修复机制，可能增强其他原因引起的肺损伤的效果，包括脓毒症、放疗、既往肺损伤和其他药物^[8-12]^。EGFR-TKIs也可能通过降低EGFR磷酸化和同步再生的上皮增生来促进潜在的肺纤维化^[13]^。

目前国际上对于奥希替尼诱发ILD后再挑战的风险和获益尚无共识。参考EGFR-TKIs不良反应管理专家共识^[14]^，EGFR-TKIs相关ILD可以分为4级，其中2级-4级的肺毒性建议停用EGFR-TKIs，并开始氧疗及激素治疗，但对于EGFR-TKIs能否再挑战及其时机尚无共识。2018年美国临床肿瘤学会（American Society of Clinical Oncology, ASCO）关于免疫治疗相关不良反应管理指南^[15]^，对于3级-4级免疫相关肺毒性建议终生停用免疫检查点抑制剂，而2级免疫相关肺毒性在应用激素治疗降级为1级免疫相关肺毒性后可再次应用免疫检查点抑制剂治疗。特别注意的是激素抵抗的肺炎患者，与英夫利息单抗相比，静脉免疫球蛋白可降低死亡率（43% *vs* 100%）^[16]^。表 1总结了目前文献报道的20例奥希替尼再挑战病例^[17-26]^，从中我们可以看到与免疫治疗相关肺毒性类似，对于1级-2级奥希替尼相关ILD，待临床症状改善后，奥希替尼再挑战相对安全，对于3级-4级患者再挑战风险较高，但权衡利弊后也有再挑战成功的案例。在我们的病例中，患者在皮质类固醇治疗下快速恢复，同时停用奥希替尼后病情进展。在权衡尚无其他有效治疗方案时患者的风险和受益，并获得患者及其家人的知情同意后，再次给予该患者奥希替尼（80 mg, *qd*），同时给予口服泼尼松，该患者持续16个月以上无病情进展。与既往文献^[17-26]^相比，我们的患者取得了非常显著的无进展生存。

**表 1:** 奥希替尼再挑战文献总结 Literature review of osimertinib rechallenges

Cases	Age/Gender	*EGFR* mutation	Onset of ILD	ILD grade	Corticosteroid (dose)	Osi cessation period	Recurrence of ILD	References
1	32/M	Del 19 T790M	4.5 mon	NA	Yes	NA	No	[14]
2	82/M	Del 19 T790M	8 mon	4	Yes	2 mon	No	[15]
3	60/M	Del 19 T790M	6 wk	3	Yes	NA	Yes	[15]
4	69/F	L858R T790M	55 d	NA	PSL 10 mg/d	15 d	No	[16]
5	32/M	L858R T790M	135 d	NA	Yes	2 mon	No	[17]
6	75/F	Del 19 T790M	64 d	2	PSL 0.5 mg/kg	26 d	No	[18]
7	62/M	Del 19 T790M	82 d	2	Yes	14 d	No	[19]
8	38/M	L858R T790M	31 d	2	No	NA	No	[20]
9	75/F	Del 19 T790M	6 mon	2	Yes	NA	No	[21]
10	73/F	L858R	3.3 mon	1	No	3.2 mon	No	[22]
11	56/M	L858R	1.0 mon	2	PSL 1 mg/kg	1.4 mon	Yes	[22]
12	45/F	Del 19	2.8 mon	2	PSL 1 mg/kg	6.3 mon	No	[22]
13	72/F	Del 19	5.9 mon	1	No	1.4 mon	No	[22]
14	64/F	Del 19	7.7 mon	1	No	14.0 mon	No	[22]
15	39/F	L858R	1.8 mon	2	No	6.2 mon	No	[22]
16	72/F	Del 19	5.6 mon	2	PSL 1 mg/kg	14.7 mon	No	[22]
17	71/F	Del 19	2.7 mon	1	No	15.2 mon	No	[22]
18	58/F	Del 19	8 wk	NA	PSL 1 mg/kg	4 wk	No	[23]
19	65/F	Del 19 T790M L858R	7 wk	NA	PSL 0.5 mg/kg	6 wk	No	[23]
20	64/M	L858R T790M	9 mon	3	PSL 1 mg/kg	14 wk	No	[23]
21	64/M	Del 19	60 d	2	Yes	5 mon	No	Present case
EGFR: epidermal growth factor receptor; ILD: interstitial lung disease; Osi: Osimertinib; PSL: Prednisolone; NA: not available; F: female; M: male.								

总之，我们的病例表明，在某些病例中，奥希替尼诱导的1级-2级ILD恢复后，再次给予奥希替尼（联合皮质类固醇）可能是可行的和获益的，其结果与既往的文献报道^[17-26]^一致。尽管如此，在发生ILD的情况下，是否选择奥希替尼再挑战需要慎重权衡患者的风险及获益，必要时可请多学科会诊介入，希望本案例能为部分患者的临床决策提供参考。
